# Pandemic Influenza: The Inside Story

**DOI:** 10.1371/journal.pbio.0040050

**Published:** 2006-02-14

**Authors:** Henry Nicholls

## Abstract

Recent developments in the molecular genetics of influenza provides clues into how avian flu - H5N1 - could find a way to jump easily from human to human and cause a pandemic

At about midnight on 7 September 2004, a mother arrived at her 11-year-old daughter's bedside in a provincial hospital in Thailand. She sat down, hugged and kissed her child, and wiped secretions from her mouth. Little did she know that her daughter's breathing difficulties were caused by the deadly H5N1 avian influenza virus. The next day, the girl was dead and, nearly two weeks later, so too was the mother, making a strong case for the virus passing from one human to another [1].

Until the influenza A virus H5N1 finds a way to do this with greater efficiency, it will not be causing a pandemic. Yes, it has managed the leap from birds to humans, and yes, it has killed about half of those unfortunate enough to catch it. But we need only be really worried if the virus somehow stumbles upon a genetic combination that allows it to jump easily from human to human. Only then will we face the pandemic that the world's media would have us believe is imminent.

So do we have any idea how avian H5N1 might acquire the deadly property of human-to-human transmission? Recent developments in the molecular genetics of influenza A could provide some answers. At the very least, they are laying important groundwork that should help tame the impact of a pandemic version of H5N1, should it appear.

## Rapid Evolution

There are three kinds of influenza: A, B, and C. Influenza B and C aren't much to worry about, at most causing minor illness. The influenza A viruses, by contrast, are highly variable and so have the potential to outwit the human immune system and cause a pandemic (Box 1).

**Figure 1:**
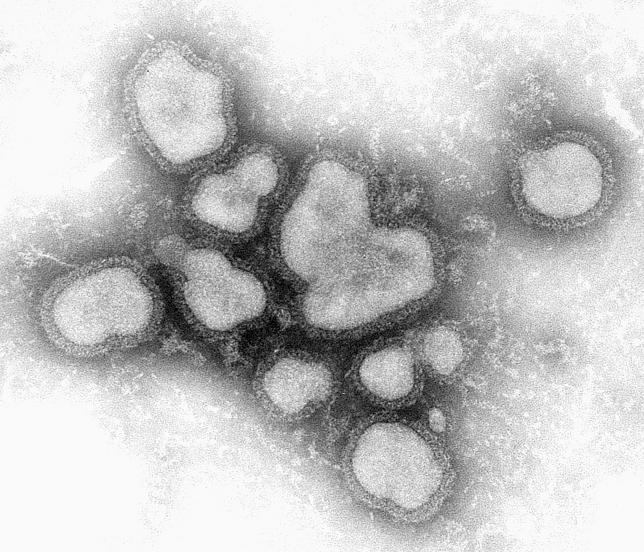
Influenza A Virus (Image: CDC/F. A. Murphy)

A year ago, there were only seven complete sequences of the common influenza A subtype H3N2 mapped out in public-domain databases. By the end of 2005, there were more than 500. This immense molecular leap is largely due to the Influenza Genome Sequencing Project, a partnership between the US National Institute of Allergy and Infectious Diseases and collaborators around the world (Figure 1).

This has radically changed what we know about the evolution of influenza A viruses, says Steven Salzberg of The Institute for Genomic Research at the University of Maryland, Rockville, Maryland, United States, and one of the scientists involved in the sequencing project. This research gives an in-depth insight into the evolution of the H3N2 subtype in New York state between 1999 and 2004, findings that almost certainly apply to other influenza viruses, including avian H5N1 [2].

RNA-based organisms such as influenza viruses present a special problem for the would-be sequencer: the RNA must first be converted to DNA. This can be achieved by calling upon the talents of reverse transcriptase, an enzyme that retroviruses such as HIV use to churn out DNA from an RNA template. The challenge is to get the reverse transcriptase to bind to the viral RNA so it can set about its work.

“It's a chicken and egg problem,” Salzberg says. The reverse transcriptase needs to be instructed where to bind to the RNA and how much of it to copy. But developing primers to do this requires knowledge of the RNA sequence, the very unknown you're trying to find. Salzberg and his colleagues on the Influenza Genome Sequencing Project managed to cobble together primers based on existing fragments of H3N2 that had already been sequenced. Armed with this valuable set of primers, they have sequenced several hundred H3N2 human influenza isolates from the New York area.

The major finding, first published in *PLoS Biology* and recently in *Nature*, is that there is surprising genetic variation within a single influenza subtype that would not be detected without this whole-genome approach [2,3]. Although all New York isolates they scrutinized were H3N2 viruses, phylogenetic analysis based on the entire genetic sequence reveals three distinct subpopulations, some more sensitive to vaccine than others. “In the 2003–2004 flu season, a new dominant strain emerged from one of these subpopulations against which the vaccine was largely ineffective,” Salzberg says. This suggests that there is important genetic variation that is being missed by tagging these variants with the same “H3N2” label. “We may need a new way of categorizing these viruses that will reflect these differences,” he says. The Influenza Genome Sequencing Project is now working on putting together a set of primers designed for H5N1, Salzberg says.

## Generating Variation

There are thought to be two main mechanisms through which such genetic variation is generated. First, single amino acid changes are frequently introduced during virus replication. Second, if two different viruses infect the same individual, they can exchange one or more genetic segments, a process known as reassortment.

Salzberg's analysis demonstrates both of these processes in the New York samples. This helps to explain why influenza is such a slippery customer and why a vaccine that works one year will be ineffective the next: frequent mutation allows the virus to keep one step ahead of the human immune system, and reassortment means that sometimes it will take a sudden sideways leap, completely outwitting our defenses.

**Figure 2:**
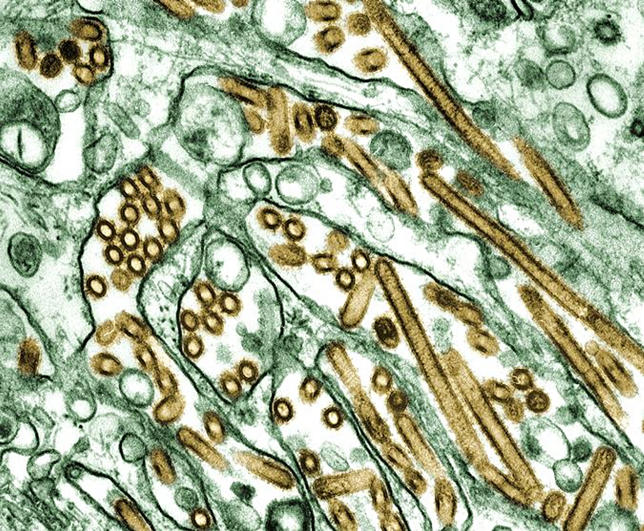
Transmission Electron Micrograph of Avian Influenza A H5N1 Viruses (Gold) (Image: C. Goldsmith, Public Health Image Library [CDC])

These tricks of the influenza A virus trade—frequent mutation and reassortment—appear to be generating even greater variation in the current bête noire, H5N1 (Figure 2). In 2004, phylogenetic analysis of a handful of H5N1 strains confirmed that a series of reassortment events had given rise to several competing forms of the virus [4].

This may account for the appearance of H5N1 in an alarmingly diverse range of species. The 2004 family tree indicates that the H5N1 strain dominant in southeast Asia originated in domestic ducks. It has, however, now become endemic in poultry across Asia, found its way into countless species of wild bird, and even turned up in mammals such as pigs, cats, leopards, tigers, and, of course, humans. The concern is that one of the many genetic incarnations of this virus will hit upon what it takes to be transferred from person to person.

## Pandemic Mutations

The 20th century saw three influenza pandemics (Box 2). Jeffrey Taubenberger and his co-workers at the Armed Forces Institute of Pathology in Washington, D.C., United States, want to know if there are molecular lessons that can be learned from these flu strains from history: how did these strains evolve in the first place, is there anything about their genetic makeup that enabled them to cause a global outbreak of disease, and does H5N1 show any of the same killer features?

Molecular work in the 1970s and 1980s established that the 1957 and 1968 pandemic strains were the result of reassortment events. In 1957, the human influenza then “doing the rounds” acquired three genetic segments from an avian source, probably Eurasian wildfowl. This radical microbial innovation left the world's human population vulnerable. In 1968, another reassortment mixed things up yet again.

“In the 2003–2004 flu season, a new dominant strain emerged....against which the vaccine was largely ineffective.”

**Figure 4:**
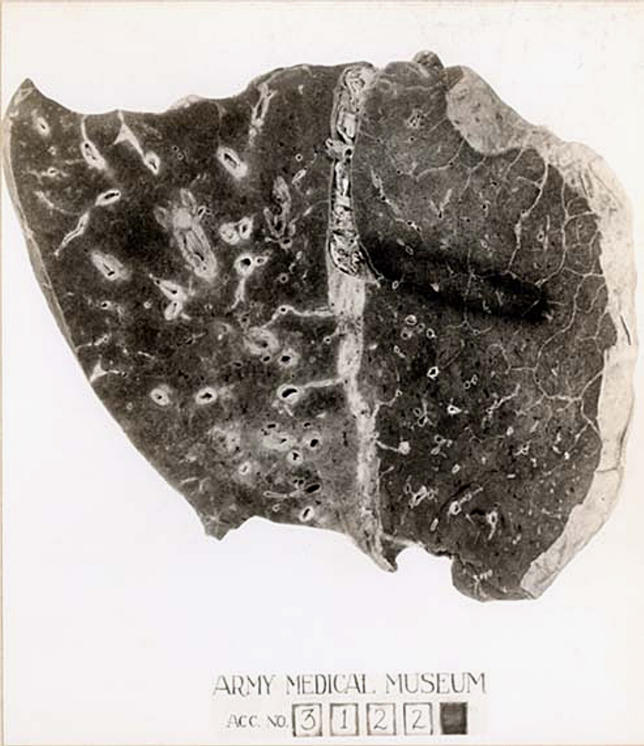
Lung from Influenza Victim Similar to That Used to Extract RNA from the 1918 Killer Strain (Image: courtesy of the National Museum of Health and Medicine, Armed Forces Institute of Pathology, Washington, D.C., United States.)

The origin of the 1918 pandemic strain, by contrast, has been harder to crack. Nearly a decade ago, Taubenberger and his colleagues made a real breakthrough; they found isolates of the 1918 pandemic virus in the formalin-fixed, paraffin-embedded lungs of an American serviceman [5] (Figure 4). They subsequently retrieved further samples of this deadly virus from a second soldier and also from a flu victim exhumed from a frosty mass grave in Alaska. Since then, they carefully sequenced one gene after another until they completed the task last year [6].

The complete sequence of the 1918 pandemic strain reveals a key finding. Each segment is more similar to avian viruses than to segments from any human strains. This suggests that it did not emerge through reassortment like the 1957 and 1968 subtypes, but evolved directly via mutation from an avian virus. Taubenberger is hoping to find a bit of preserved tissue that contains a precursor to the 1918 virus. “I would expect to find a virus very similar to 1918 absent a few critical mutations that might be associated with human adaptation,” he predicts.

Although this research on the 1918 virus suggests that a pandemic version of H5N1 could emerge directly from an avian H5N1, Taubenberger is more concerned about genetic exchange between an avian and a human virus. “I would still bet on reassortment as the easiest mechanism to gain human-to-human transmissibility,” he says.

In spite of the revelation that a pandemic strain can emerge through at least two different mechanisms, there may still be telling similarities between the three 20th-century pandemics. There are ten key mutations in the polymerase genes common to 1918, 1957, and 1968 that could allow adaptation to mammals, Taubenberger says. At the moment, it's not entirely clear what these mutations are actually doing, but some of them could be important.

The set of H5N1 primers being developed by the Influenza Genome Sequencing Project should allow rapid sequencing of emerging strains of avian H5N1s to see if any of these key changes have occurred. This could give advance warning of the strains that pose the greatest threat, says Robert Belshe, professor of medicine, pediatrics, and molecular microbiology at Saint Louis University School of Medicine, St. Louis, Missouri, United States [7]. “We can get a handle on when some of these genetic changes will be acquired, and that may guide us to which virus may emerge as the next pandemic virus,” he says.

Of course, the next pandemic need not arise as a result of these same mutations, Taubenberger says. “A virus might adapt in multiple ways, unique for each event.” Nevertheless, there might be certain crucial mutations that will always be required, he suggests. At least three of the ten polymerase mutations have already occurred in H5N1s that infected humans, although not together in the same isolate. This suggests that H5N1 must undergo further genetic innovation before it can go pandemic. “There are still a number of changes that would have to happen in order for the virus to replicate efficiently and spread in people,” Belshe says.

## Prevention

Meanwhile, H5N1 continues to advance around the globe. Whilst it remains a problem firmly rooted in Asia, its appearance in Turkey, Romania, and Croatia last year raises the possibility that birds migrating to Africa this winter will have taken it with them. The close proximity between people and animals in eastern African countries creates another ideal breeding ground for the virus, says Joseph Dromenech, chief veterinary officer for the Food and Agriculture Organization of the United Nations. “Surveillance and capability to respond immediately after the detection of an outbreak is insufficient,” he says. Surveillance is important because culling infected birds dramatically reduces the chance that humans will come into contact with the virus and, therefore, the opportunity for avian H5N1 to reassort with human influenza.

Governments have also begun stockpiling antiviral drugs, which can block replication of most influenza A viruses or prevent their release from infected cells. Individuals have even gone in search of antivirals. In October of last year, the Internet auction giant eBay removed all listings for one antiviral from its British site as bids spiralled to more than £100 for a single dose.

**Figure 5:**
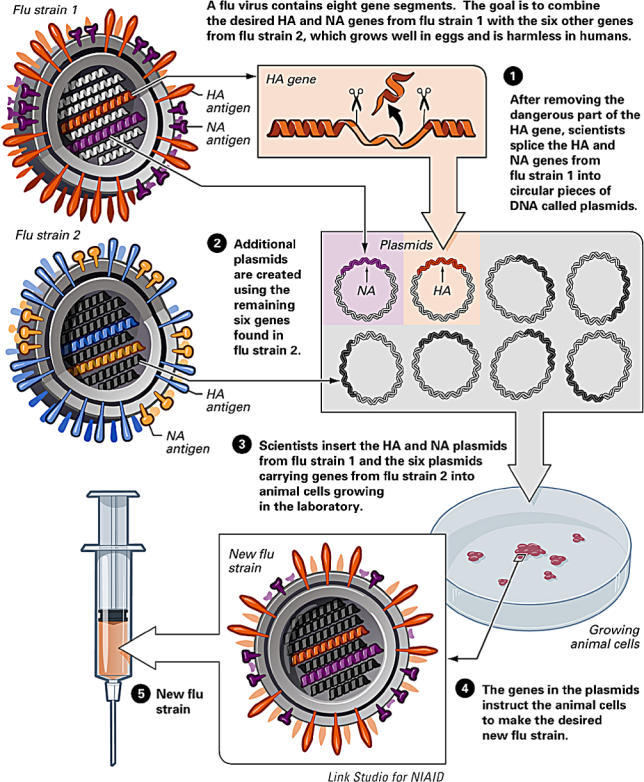
The Mechanics of Reverse Genetics (Image: courtesy of US National Institute of Allergy and Infectious Diseases.)

A more realistic means of coping with a pandemic is by vaccination. In the US, clinical trials are already under way to test vaccines against a strain of H5N1 made safe through a technique called “reverse genetics” (Figure 5). This involves stitching together DNA plasmids, each containing a single influenza gene. “You can design your own virus; therefore you can design your own vaccine,” says George Brownlee, professor of chemical pathology at Oxford University, United Kingdom, whose group pioneered this technique in the late 1990s [8].

Such trials should help confirm the safety of vaccination and determine the dosage and the number of doses needed to achieve effective immunity, Belshe says. Hopefully, antibodies raised from this vaccine will recognise the surface antigens on a pandemic influenza, giving some protection as researchers rush to decode the sequence of a pandemic strain and manufacturers work overtime to produce a vaccine against it. “Until we know what exact strain is going to occur and cause a pandemic, we really can't make and stockpile vaccine,” Belshe says.

So reverse genetics is also being used to try to second-guess the virus' next move and explore which reassorted viruses have what it takes to cause a pandemic. These experiments are based on the assumption that if avian H5N1 reassorts with a human influenza, it will most likely do so with human H3N2. Virologists at the US Centers for Disease Control and Prevention (CDC) in Atlanta, Georgia, are mixing genes from these two strains to find out which combinations are capable of infecting mammalian cells and hence pose a threat to humans.

This approach is useful for determining the virulence and pathogenicity of different hypothetical reassortant strains, says Albert Osterhaus, professor of virology at Erasmus Medical Center, Rotterdam, The Netherlands. He is carrying out similar experiments working with another threatening avian subtype—H7N7—rather than H5N1. However, he points out, there is no way of finding out how efficiently these lab-confined strains will pass from human to human. We will learn this only once a pandemic is under way.

## The Near Future

Molecular cunning is clearly an important tool in preparing ourselves for an impending pandemic. The more we learn about the trickery used by influenza strains from history, the better placed we will be to determine what molecular changes are needed for a virus to pass from human to human. Armed with this knowledge, we may be able to anticipate the evolution of strains of avian influenza in the near future, helping to combat the next flu pandemic when (not if) it occurs.

**Box 1. The Background to Influenza A**

Influenza A is made up of a protein coat or capsid that houses the viral genome, a single strand of RNA split into eight segments, each carrying a single gene. One of these encodes hemagglutinin (HA), a surface antigen that the virus uses to bind to and break into host cells. Another gene produces a second surface antigen, neuraminidase (NA), which helps newly formed virus escape to infect other cells.

The host immune system targets HA and NA, subjecting them to strong selective pressure. Genetic mutation, subtly altering the antigenic properties of these two target proteins, is sometimes referred to as antigenic *drift*. Antigenic *shift*, by contrast, is a major change in the antigenic properties of these two proteins caused by reassortment of one or more of the 16 known HA subtypes (H1 to H16) or the nine NA subtypes (N1 to N9) that circulate in wild birds.

Humans have been exposed to only H1, H2, and H3 viruses in the recent past. Consequently, a virus with an unfamiliar subtype, such as H5N1, will go undetected by the immune system of everyone alive today.

The six other genes that make up the influenza genome have received less scrutiny, but there is increasing evidence that they play an important role in adaptation of virus to host.

**Box 2. Outbreaks in History**

**Figure 3:**
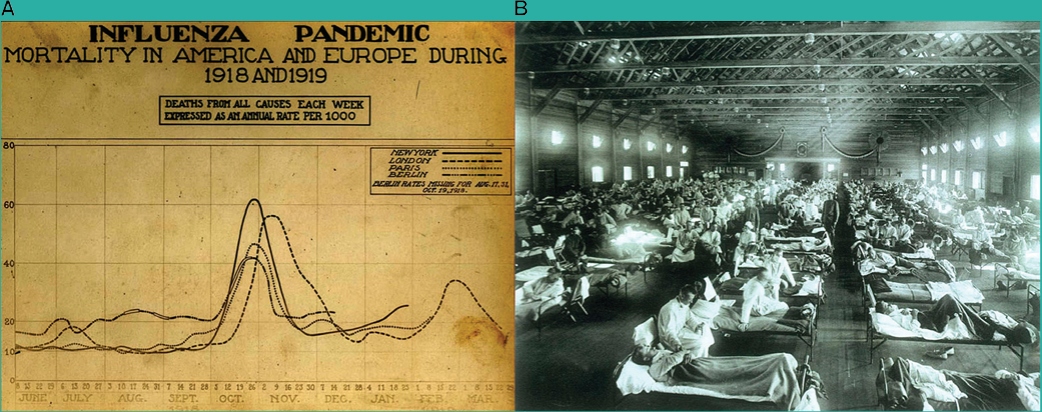
The Spanish Influenza (A) Chart showing mortality from the 1918 influenza pandemic in the US and Europe. (B) Emergency military hospital during influenza epidemic, Camp Funston, Kansas, United States. (Image: courtesy of the National Museum of Health and Medicine, Armed Forces Institute of Pathology, Washington, D.C., United States.)

In the past century, there were three influenza pandemics. The “Spanish influenza” of 1918 caused by the H1N1 subtype is estimated to have hit nearly a third of the world's population (Figure 3A). Conditions at the end of World War I may have contributed to the mortality; in just one year, it killed more than 40 million people (Figure 3B). The “Asian influenza” of 1957 resulted from a reassortment event, generating a new subtype: H2N2. There was little or no pre-existing immunity to this reassorted virus, and it is believed to have caused more than 2 million deaths. Eleven years later, further reassortment gave the prevailing human influenza a new surface protein, resulting in H3N2. This subtype killed about a million people and is currently the dominant subtype of human influenza.
